# Comparison of acute and prolonged effects of short-term foam rolling and vibration foam rolling on the properties of knee extensors

**DOI:** 10.5114/biolsport.2024.129488

**Published:** 2023-09-21

**Authors:** Kazuki Kasahara, Andreas Konrad, Riku Yoshida, Yuta Murakami, Shigeru Sato, Ryoma Koizumi, Ewan Thomas, David G Behm, Masatoshi Nakamura

**Affiliations:** 1 Institute for Human Movement and Medical Sciences, Niigata University of Health and Welfare, Niigata, Japan; 2 Institute of Human Movement Science, Sport and Health, University of Graz, Graz, Austria; 3 Department of Physical Therapy, Niigata University of Health and Welfare, Niigata, Japan; 4 Sport and Exercise Sciences Research Unit, Department of Psychology, Educational Science and Human Movement, University of Palermo, Palermo, Italy; 5 School of Human Kinetics and Recreation, Memorial University of Newfoundland, St. John’s, Newfoundland and Labrador, Canada; 6 Faculty of Rehabilitation Sciences, Nishi Kyushu University, 4490-9 Ozaki, Kanzaki, Saga, 842-8585, Japan

**Keywords:** Range of motion, Countermovement jump, Warm-up, Tissue hardness, Pain pressure threshold

## Abstract

Recently, Foam Rolling (FR) and Vibration Foam Rolling (VFR) have attracted attention in sports and rehabilitation fields. Previous studies have shown that FR and VFR acute interventions effectively increase the range of movement (ROM) and decrease tissue hardness. For application to sports and rehabilitation, it is necessary to compare the acute and prolonged effects of short duration FR and VFR. Therefore, this study aimed to compare and investigate the acute and prolonged (15 minutes) effects of short duration (30-s) FR and VFR interventions on knee extensors. The subjects were 14 male university students (22.4 ± 1.0 years old), in which the knee extensors of the dominant leg were tested. In a cross-over trial, 30-s of FR or VFR were performed with 2-s rolling of the anterior thigh (15 rolls). The frequency of VFR was 35 Hz. Measurements included knee flexion ROM, pain pressure threshold (PPT), tissue hardness, and countermovement jump height. The results of this study showed no interaction effects for all variables, but main time effects were observed for knee flexion ROM, PPT, and tissue hardness. Post-hoc tests showed that knee flexion ROM increased up to 10 minutes after the intervention. PPT significantly increased, and tissue hardness significantly decreased up to 15 minutes after intervention. This study showed that 30-s FR and VFR interventions effectively increased ROM, PPT, and decreased tissue hardness. The effects were prolonged up to 10–15 minutes after the intervention. The results of this study show no advantage of VFR over FR with acute short-term interventions.

## INTRODUCTION

Foam rolling (FR) has recently attracted attention in sports and rehabilitation fields [1]. Previous studies on the effect of FR have reported an increase in ROM [2–4] and a decrease in tissue hardness [2, 5]. Moreover, vibration foam rolling (VFR), in which FR is provided with a vibration function, has been suggested to increase ROM [6] and decrease tissue hardness [7] to a greater extent than FR. Furthermore, a previous study [8] compared the effects of FR and VFR interventions on delayed onset muscle pain induced by exercise with induced muscle damage. They showed that VFR was more effective than FR in improving pain and hip extension ROM. In addition, VFR for older women has been reported to be effective in improving senior fitness performance when incorporated as a warm-up [9] as well as an increase in shoulder flexibility when combined with static stretching [10]. On the other hand, FR and VFR intervention for the ankle plantar flexors was reported to be effective in increasing ROM on the nonintervention side to the same extent [7]. Also, FR and VFR interventions have not been reported to decrease muscle strength or performance [11, 12]. Therefore, FR and VFR interventions that maintain muscle strength and increase ROM are currently being employed in various sports and rehabilitation fields.

A previous study suggested that FR aimed at increasing ROM requires interventions of 90-sec or longer. In a previous study investigating the effects of a 180-second FR and VFR intervention on knee extensors [2], an increase in knee flexion ROM was prolonged for at least 30 minutes after the intervention with both FR and VFR interventions. However, since static stretch interventions in sports fields have been reported to be mostly less than 20 seconds [13], it is essential information for athletes and coaches about the effect of a more practical duration, i.e., short-duration FR and/or VFR interventions. Regarding the effect of a short-term FR intervention, a previous study investigating the acute effect of a 5-second FR intervention on ham-strings showed a significant increase in the Sit and Reach test without loss of muscle strength [14]. However, the prolonged effects of short-term FR interventions are unknown. Moreover, VFR may have a greater effect than FR [6], and may also have a greater acute effect, even for short-term interventions. Furthermore, in view of its application in sports and rehabilitation fields, it is necessary to compare the acute and prolonged effects of short-term FR and VFR interventions. Also, FR intervention has been suggested to have a dose-response relationship [14]. In this study, the duration of the intervention is set shorter than in previous studies [2], thus we expected that the prolonged effect will also be shorter than in the previous study. Hence, we investigated the prolonged effect of FR and VFR up to 15 minutes after the intervention. Thus, this study was designed to compare and examine the acute and prolonged effects of short-term FR and VFR interventions on knee extensors. Based on previous studies [6, 7], we hypothesized that the effects of increased ROM and decreased tissue hardness would be greater in VFR compared to FR because the vibration function would affect mechanoreceptors [12, 15]. Previous studies [12, 16] have reported that FR/VFR interventions do not adversely affect muscle strength or jump performance. Therefore, we hypothesized that a short-term intervention would not alter CMJ height.

## MATERIALS AND METHODS

### Experimental set-up

**FIG. 1:**
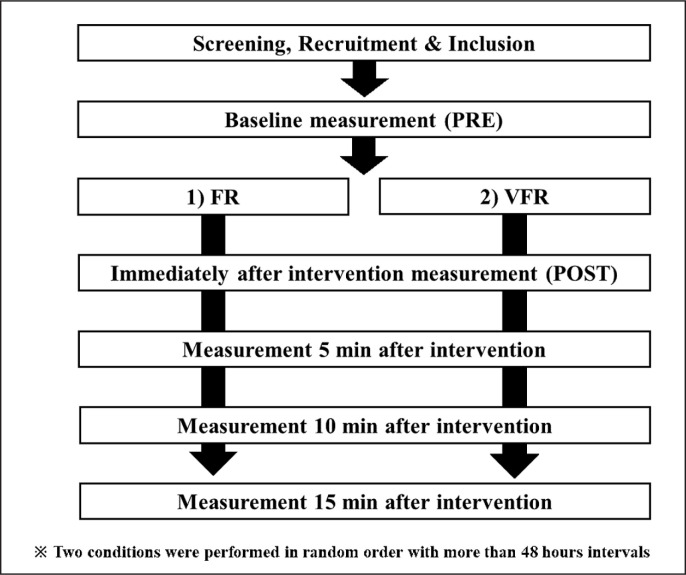
The experimental set-up FR: foam rolling, VFR: vibration foam rolling.

A randomized repeated measures experimental design was used to compare the acute and prolonged effects of short-term FR and VFR. The participants were instructed to visit the laboratory twice with a ≥ 48 h break. They performed the following two conditions, 30 seconds of FR and VFR (Figure 1). The FR and VFR were performed with a 2-second rolling intervention from proximal to distal to proximal and back to proximal of the target muscle. In both conditions, the 30-second intervention involved 15 rolls of the knee extensors of the dominant leg. Measurement points were pre-intervention (PRE), immediate post-intervention (POST), and 5-, 10-, and 15 minutes after the intervention. Outcome measures were tissue hardness, pain pressure threshold (PPT), knee flexion ROM, and CMJ evaluated in this order. Since dynamic activity such as a CMJ can affect ROM and stretching can impact PPT and muscle tissue hardness [17–19], the order of tests was not randomized. The reproducibility of the measured items has been confirmed in our previous study [20] with good to excellent reliability in the present study as well (see results section).

### Participants

Fourteen healthy, recreationally active males were enrolled (mean ± SD: age, 22.4 ± 1.0 years; height, 170.7 ± 4.8 cm; weight, 67.6 ± 9.2 kg). Individuals with a history of neuromuscular disease and musculoskeletal injury involving the lower extremities were excluded. The required sample size for a repeated-measures two-way analysis of variance (ANOVA) (effect size = 0.25 [large when considering interaction effects for 2-way ANOVAs], α error = 0.05, and power = 0.95) based on our previous study’s ROM results [21] using G* power 3.1 software (Heinrich Heine University, Dusseldorf, Germany) was more than 9 participants.

For the study, participants were fully informed about the procedures and aims, after which they provided written informed consent. The study complied with the requirements of the Declaration of Helsinki and was approved by the Ethics Committee of the Niigata University of Health and Welfare, Niigata, Japan (Procedure#18615).

### Outcome assessments

#### Knee flection ROM

Each participant was placed in a side-lying position on a massage bed with the hips as well as the knee of the non-dominant leg flexed at 90° to prevent pelvic movements [22, 23]. A licensed physical therapist, the investigator, brought the dominant leg to full knee flexion with the hip joint in a neutral position. A goniometer (MMI universal goniometer Todai 300 mm, Muranaka Medical Instruments, Co., Ltd., Osaka, Japan) was used to measure knee flexion ROM. This was measured three times at each measurement point, and the average values at each measurement point were used for analysis.

### Pain pressure threshold (PPT)

PPT measurements were conducted in the supine position using an algometer (NUTONE TAM-22 (BT10); TRY-ALL, Chiba, Japan). The measurement location was set at the midway of the distance between the anterior superior iliac spine and the dominant side’s superior border of the patella for the rectus femoris muscle. With continuously increasing pressure, the soft tissue in the measurement area was compressed with the metal rod of the algometer. The participants were instructed to immediately press a trigger when pain, rather than just pressure, was experienced. The value read from the device at this time point (kilograms per square centimetre) corresponded to the PPT. In each condition, PPT was measured three times at each measurement point, and the mean value at each measurement point was used for further analysis.

### Tissue hardness

Tissue hardness was measured using a portable tissue hardness meter (NEUTONE TDM-N1; TRY-ALL Corp., Chiba, Japan). The participant’s measurement position and posture were similar to PPT measurements. This tissue hardness meter measured the penetration distance until a 14.71 N (1.5 kgf) pressure was reached [24]. The participants were instructed to relax while tissue hardness was measured three times at each measurement point, and the mean value at each measurement point was used for further analysis.

### Unilateral Countermovement Jump (CMJ) height

Unilateral CMJ height was calculated from flight time using a contact mat (Jump mat system; 4Assist, Tokyo, Japan). The participants started with the foot of the dominant leg on the mat with their arms crossed in front of their chest. The participants were instructed to dip quickly (eccentric phase) from this position, reaching a self-selected depth to jump as high as possible in the next concentric phase. Landings were performed on both feet. The knee of the non-involved leg was held at approximately 90° flexion. After three familiarization trials, three maximal unilateral CMJ were conducted at both PRE and POST in each condition, and the average of the three trials was used for further analysis [20].

### Foam rolling (FR), Vibration Foam Rolling (VFR) intervention

The participants were instructed on how to use the foam roller (Stretch Roll SR-002, Dream Factory, Umeda, Japan) by a physical therapist. For familiarization, they were allowed to practice using the foam roller three to five times on the non-dominant leg (non-intervention leg) immediately before the FR intervention to verify that the participants were able to perform the FR intervention at the specified velocity and location. Based on the Behm et al. [4] recommendations, FR and VFR involved 1 set of 30-seconds with 2-second cycles (defined as one distal rolling movement followed by one proximal rolling movement). A metronome (Smart Metronome; Tomohiro Ihara, Japan) was used for control. The participants were asked to place as much body mass on the roller as tolerable.

### Statistical analysis

SPSS (version 28.0; IBM Corp., Armonk, NY, USA) was used for the statistical analysis. To verify the consistency of PRE values, PRE values were tested between FR and VFR conditions using a paired t-test. For all the variables, a two-way repeated-measures ANOVA using two factors (test time [PRE vs. POST vs. 5 minutes vs. 10 minutes vs. 15 minutes] and conditions [FR vs. VFR]) was used to analyze the interaction and main effects. Classification of effect size (ES) was set where $\eta_{\text{p}}^{2}$ < 0.01 was considered small, 0.02 – 0.1 was considered medium, and more than 0.1 was considered to be a large effect size [25]. Where appropriate, post-hoc analyses were performed using multiple comparison tests with Bonferroni correction to determine differences between PRE, POST, 5, 10, and 15 minutes. Also, we calculated the effect sizes (Cohen d) on the PRE value. Cohen d of 0.00–0.19 was considered trivial, 0.20–0.49 was small, 0.50–0.79 was moderate, and ≥ 0.80 was large [26]. The significance level was set to 5%, and all the results are shown as mean ± SD.

## RESULTS

### Comparison between PRE values among the two conditions

There were no significant differences in all PRE variables between the two conditions. We calculated the coefficient of variation (CV) and intraclass correlation coefficient (ICC) from these data. The CVs of measurements for knee ROM, PPT, tissue hardness, and CMJ height were 0.8 ± 0.7%, 8.5 ± 6.0%, 6.6 ± 4.3%, and 2.7 ± 1.5%, respectively, and the ICC (1,2) for measurements were 0.706, 0.805, 0.737, and 0.958, respectively.

### Changes in knee flexion ROM, PPT, tissue hardness, and CMJ height

**FIG. 2:**
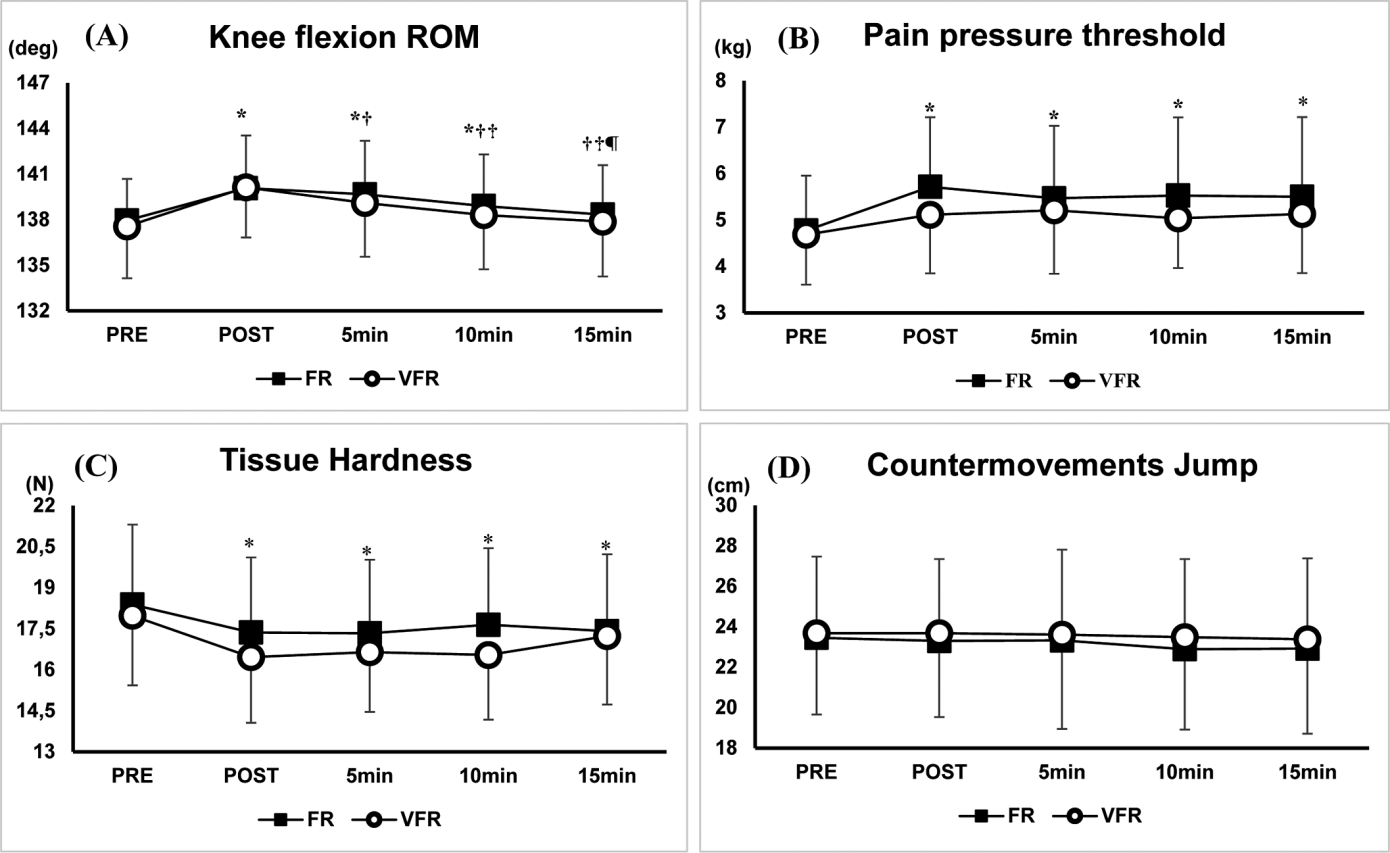
Changes in knee flexion range of motion (ROM), pain pressure threshold, Tissue Hardness, and countermovement jump height after 30 seconds foam rolling (FR) and vibration foam rolling (VFR) intervention (*: Significant difference (p < 0.01) from PRE, †: Significant difference (p < 0.01) from POST, ‡: Significant difference (p < 0.01) from 5 minutes after the intervention, ¶: Significant difference (p < 0.05) from 10 minutes after the intervention).

There were no significant interaction effects for all variables (knee flexion ROM: F = 0.7, p = 0.56, $\eta_{\text{p}}^{2}$ = 0.03, PPT: F = 1.0, p = 0.39, $\eta_{\text{p}}^{2}$ = 0.04, tissue hardness: F = 1.9, p = 0.12, $\eta_{\text{p}}^{2}$ = 0.07, CMJ height: F = 0.2, p = 0.94, $\eta_{\text{p}}^{2}$ = 0.01) (Figure 2). There were significant (p < 0.01) main effects of test time with knee flexion ROM (F = 37.4, p < 0.01, $\eta_{\text{p}}^{2}$ = 0.61), PPT (F = 7.9, p < 0.01, $\eta_{\text{p}}^{2}$ = 0.25), and tissue hardness (F = 14.6, p < 0.01, $\eta_{\text{p}}^{2}$ = 0.38), but there was no significant main effect of test time for CMJ height (F = 1.3, p = 0.26, $\eta_{\text{p}}^{2}$ = 0.05). The post-hoc test results showed that knee flexion ROM was significantly (p < 0.05) higher at POST (FR: d = 0.64, 1.5 ± 0.8%, VFR; d = 0.67, 1.9 ± 1.5%), 5 (FR: d = 0.52, 1.2 ± 0.9%, VFR: d = 0.43, 11.1 ± 0.9%) and 10 minutes (FR: d = 0.33, 0.7 ± 0.8%, VFR: d = 0.22, 0.5 ± 0.6%) after the intervention compared to PRE. However, ROM returned to baseline values 15 minutes after the intervention (FR: d = 0.15, 0.3 ± 0.6%, VFR: d = 0.08, 0.2 ± 0.6%). Knee flexion ROM was significantly lower after 5, 10, and 15 minutes after the intervention compared with POST (p < 0.01) and was also lower after 10 and 15 minutes compared with 5 minutes after the intervention (p < 0.01). Moreover, it was significantly lower after 15 minutes after the intervention compared to 10 minutes after the intervention (p < 0.05). PPT was significantly (p < 0.05) higher at POST (FR: d = 0.59, 20.9 ± 18.8%, VFR: d = 0.45, 9.9 ± 16.3%), 5 (FR: d = 0.40, 15.2 ± 20.2%, VFR: d = 0.41, 11.7 ± 18.8%), 10 (FR: d = 0.42, 16.1 ± 2.20%, VFR: d = 0.29, 9.0 ± 16.3), and 15 minutes (FR: d = 0.42, 15.0 ± 18.5%, VFR: d = 0.33, 10.1 ± 15.0%) after the intervention compared to PRE. Tissue hardness was significantly (p < 0.05) lower at POST (FR: d = 0.29, -5.4 ± 5.0%, VFR: d = 0.53, -8.2 ± 7.2%), 5 (FR: d = 0.33, -5.6 ± 5.1%, VFR: d = 0.54, -7.0 ± 6.9%), 10 (FR: d = 0.25, -4.0 ± 4.4%, VFR: d = 0.58, -7.9 ± 5.5%), and 15 minutes (FR: d = 0.32, -5.3 ± 4.8%, VFR d = 0.30, -4.0 ± 6.0%) after the intervention compared to PRE.

## DISCUSSION

This study compared the acute and prolonged effects of short-term (i.e., 30-second) FR and VFR interventions on knee extensors. This study showed that both 30-second FR and VFR increased knee flexion ROM up to 10 minutes after the intervention. Also, PPT significantly increased, and tissue hardness significantly decreased up to 15 min after both FR and VFR interventions. However, no significant change in CMJ was observed. In addition, there were no significant differences between FR and VFR interventions for any measure. These results suggest that acute, short-duration FR and VFR interventions are recommended when the goal is to increase ROM without decreasing performance. To the best of our knowledge, this is the first study examining and comparing the acute and prolonged effects of short-term FR and VFR interventions.

The significant increases in knee flexion ROM were 2.14 ± 0.01°from PRE to POST, 1.56 ± 0.20° from PRE to 5 minutes, and 0.88 ± 0.21 from PRE to 10 minutes, respectively, after 30-sec FR interventions in this study. These increases in ROM were consistent with several previous studies that report moderate to large magnitude effect size [27–29] FR-induced knee flexion improvements immediately post-intervention ranging from 1.4–8.0° [29–32]. Only two FR studies have not reported an immediate significant knee flexion ROM increase with either 2 minutes of FR [33] or a combination of jogging and FR for 8 minutes [8]. By the present findings, a recent study has shown no significant difference in increased ROM between FR and VFR [7].

The significant knee flexion ROM increase up to 10 minutes in the present study is in agreement with MacDonald et al. [34], who found 10° and 8° knee flexion ROM improvements at 2- and 10-minutes post-FR intervention. However, the present findings are in contrast to Wilke et al. [5], who did not find a significant FR-induced knee flexion ROM increase immediately, 5-, or 10-minute post-test. The Wilke et al. [5] study used four repetitions of 45 seconds each at either a high or low rolling velocity, whereas the present study incorporated only one set of 30 seconds. A previous study from this lab showed no significant increase in ankle dorsiflexion ROM after 30 seconds but a significant increase in ankle dorsiflexion ROM after more than 90 seconds FR intervention [35]. This discrepancy between the previous study from this lab and the present results may vary depending on the targeted muscle. Nakamura et al. [35] investigated the acute effect of short-term FR intervention on ankle plantar flexors, but the current study investigated the acute effect of shortterm FR intervention on knee extensors. Also, Sullivan et al. [14] showed that even a 5-second FR intervention on hamstrings increased the Sit and Reach test. Thus, the FR intervention duration required to increase ROM in the target muscle may differ. Therefore, it is necessary to examine and compare the minimum FR intervention duration required to increase ROM in various muscles.

In addition, previous studies suggested that increases in stretch (pain) tolerance are involved with the increase in ROM after FR and VFR interventions [1, 7, 35, 36]. An FR intervention may reduce pain by activating either or both neural-gating mechanisms [37, 38] or the release of endorphins and enkephalins as theorized with the diffuse noxious inhibitory control mechanism [39]. The results of this study showed that the knee flexion ROM returned to the baseline value 15 minutes after the intervention. Previous studies with a 180-second intervention period [2, 3] showed that the prolonged effect of increased ROM lasted for 20–30 minutes. FR suggests a capacity-response relationship [14]. In this study, the duration of the intervention is set shorter than in previous studies [2, 3], thus it can be assumed that the prolonged effect was shorter than in the previous studies. With this study, a significant increase in PPT after shortterm FR and VFR intervention was prolonged for up to 15 minutes after both interventions. On the other hand, the significant increase in knee flexion ROM only persisted for 10 minutes after both interventions.

Tissue hardness was also significantly decreased up to 15 minutes after the intervention. A systematic review and meta-analysis examining the acute effects of FR interventions highlighted decreases in tissue hardness with the thoracolumbar fascia and quadriceps muscles [40]. The mechanism of the decrease in tissue hardness after FR intervention could be thixotropic changes (decreased tissue visco-elasticity) [15]. Moreover, previous studies showed that FR intervention increased tissue perfusion and consequently decreased tissue hardness [41, 42]. On the other hand, the previous study pointed out that VFR has a greater effect than FR due to the stimulation of mechanoreceptors by vibration [6, 15]. Kasahara et al. [2] reported that a 180-seconds VFR intervention could decrease tissue hardness longer than an FR intervention. In addition, Nakamura et al. [7] examined the effects of 180 seconds of FR and VFR interventions on the ankle plantar flexors. The results showed that only the VFR intervention decreased muscle stiffness of the medial gastrocnemius muscle, but not in the FR intervention. However, in the present study, there was no significant difference in the decrease in tissue hardness between short-term FR and VFR interventions. These results support previous studies [2, 43] that found a significant decrease in tissue hardness of the knee extensors after FR and VFR interventions. Interestingly, Kasahara et al. [2] reported that a 180-seconds VFR intervention could decrease tissue hardness longer than an FR intervention. The intervention duration in this study was only 30 seconds, suggesting that with a longer intervention time, there was a greater effect of the vibration function. The results of this study also suggest that even short-term intervention possibly induced thixotropic changes and increased blood flow, resulting in a decrease in tissue hardness.

However, there is an unclear relationship between PPT increases (stretch tolerance), and tissue hardness decreases over 15 minutes versus ROM improvements for only 10 minutes. These findings may suggest there may be other factors contributing to the increased ROM that persist for 10 minutes or less. For example, tissue hardness is affected by tissue perfusion [42], which has been implicated in increased concentrations of oxygen and carbon monoxide [44], thus tissue hardness may not directly represent muscle tissue stiffness [41]. Although decreased tissue hardness was evident for 15 minutes post-intervention, decreased muscle stiffness has been reported to be prolonged for only 5 minutes [19, 45, 46]. Whereas PPT and muscle hardness may persist for 15 minutes post-intervention, significant increases in ROM might be attributed to the contributions of a greater variety of factors such as muscle stiffness which may have shorter time courses. Future research should consider the influence and durations of the various possible ROM increasing mechanisms associated with FR and VFR.

This study showed that short-term FR and VFR interventions did not significantly change CMJ. A previous study comparing the effects of dynamic stretching and FR in tennis players reported no significant changes in CMJ [47]. On the other hand, a previous study investigating the acute effect of a 180-second FR and VFR intervention on knee extensors showed a significant increase in muscle strength after both FR and VFR interventions [43]. The acute effect of FR intervention on muscle strength and performance with FR has not been consistent, but in recent reviews, FR interventions tend to have no beneficial or harmful effect on muscle strength or performance [4, 11] or are effective when compared to stretching interventions [48]. Furthermore, a systematic review and meta-analysis [12] examining the effects of VFR interventions showed no significant effect of VFR interventions on jumping performance. Our results expanded these findings and suggested that short-term FR and VFR interventions do not change jump performance after both FR and VFR interventions.

This study has some limitations. The first is that post-intervention measurements could only be taken up to 15 minutes after the intervention, and thus, the maximum duration of these effects (increased PPT and decreased tissue hardness) are unknown. The second limitation is the frequency of FR intervention. In this study, the speed was standardized to one cycle of 2 seconds, so it is unclear whether the effect of different FR intervention velocities differs. The third limitation is the target population. This study was conducted on healthy male university students, so it is not clear whether the same effects could be obtained on athletes or female participants. The fourth limitation is no control condition in this study. A major objective of the control condition is to ensure that the testing measures did not contribute to significant changes irrespective of the intervention. However, in our previous study [2], there were no significant changes in the control condition with the same setup. Therefore, we did not adopt a control condition in this study. Although there is no control group in this study, we believe that the results of this study demonstrate the effectiveness of FR and VFR intervention. The fifth is the intervention time. This study investigated the acute and prolonged effects of FR and VFR for 30 seconds. The previous study [14] showed an increase in sit and reach score after a 5-second FR intervention. Therefore, it is necessary to examine changes in shorter periods than the FR and VFR intervention periods used in this study (i.e., 30 s).

### Practical implications

As a pre-exercise warm-up, short-term, i.e., 30-second FR or VFR intervention is recommended when the goal is to increase ROM while maintaining muscle strength. In addition, athletes and coaches need to be careful when performing the intervention because the effect of a 30-second FR/VFR intervention to increase ROM lasts for more than 10 minutes and disappears after 15 minutes. Also, in short-term interventions, vibration function does not necessarily need to be added to FR as a warm-up routine.

## CONCLUSIONS

This study compared and examined the acute and prolonged effects of short-term FR and VFR interventions on knee extensors. The results showed that 30 seconds of intervention significantly increased ROM, and the effect lasted up to 10 minutes after the intervention. The results for PPT and tissue hardness were found to change up to at least 15 minutes after the intervention. However, there was no significant effect on CMJ height. No significant differences were found between FR and VFR, suggesting that VFR is not always necessary for short-term ROM increase and muscle stiffness reduction.
